# Interventions for reducing and/or controlling domestic violence among pregnant women in low- and middle-income countries: a systematic review

**DOI:** 10.1186/s13643-019-0998-4

**Published:** 2019-04-02

**Authors:** Diksha Sapkota, Kathleen Baird, Amornrat Saito, Debra Anderson

**Affiliations:** 1 School of Nursing and Midwifery, Griffith University, Brisbane, Australia; 2 Kathmandu University School of Medical Sciences, Dhulikhel, Nepal; 3 Gold Coast University Hospital, Brisbane, Australia; 4 Women’s Wellness Research Program, Menzies Health Institute Queensland, Brisbane, Australia

**Keywords:** Developing countries, Domestic violence, Intervention, Pregnant women, Review

## Abstract

**Background:**

Domestic violence (DV) during pregnancy is recognized as a global health problem associated with serious health consequences for both the mother and her baby. Several interventions aimed at addressing DV around the time of pregnancy have been developed in the last decade, but they are primarily from developed countries. Low- and middle-income countries (LMICs) are facing both a mounting burden of DV as well as severe resource constraints that keep them from emulating some of the effective interventions implemented in developed settings. A systematic review was conducted to examine the approaches and effects of interventions designed for reducing or controlling DV among pregnant women in LMICs.

**Methods:**

Electronic databases were systematically searched, and the search was augmented by bibliographic reviews and expert consultations. Two reviewers assessed eligibility and quality of the studies and extracted data independently. The third reviewer was involved to resolve any discrepancies between the reviewers. Due to the limited number of studies and varied outcomes, a meta-analysis was not possible. Primary outcomes of this review included frequency and/or severity of DV and secondary outcomes included mental health, safety behaviours, and use of community resources. In addition, findings from the critical appraisal of studies were utilised to inform the initial draft of Theory of Change (ToC).

**Results:**

Only five studies (two randomized trials and three non-randomized trials) met the eligibility criteria. The interventions consisting of supportive counselling demonstrated a reduction in DV and an improvement in use of safety behaviours. One study has embedded the DV intervention into an existing program on human immunodeficiency virus (HIV). Limited evidence could be drawn for outcomes such as quality of life and the use of community resources.

**Discussion:**

This review attempted to address the knowledge gap by collating evidence on interventions aimed at addressing DV among pregnant women in LMICs. The development of a ToC was critical in understanding how certain activities led to the desired outcomes. This ToC can guide the design of future research and development of practice guidelines. The participatory involvement of the stakeholders is recommended to refine the current ToC to support its further development for practice.

**Systematic review registration:**

PROSPERO, CRD42017073938

**Electronic supplementary material:**

The online version of this article (10.1186/s13643-019-0998-4) contains supplementary material, which is available to authorized users.

## Background

Rocketing epidemic rates of violence against women (VAW) and its serious health consequences have gained international significance [1]. Globally, one in three women experiences violence from an intimate partner, and one of the regions witnessing its highest prevalence is the South East Asian region (37.7%) [1]. Domestic violence (DV), domestic and family violence (DFV), and intimate partner violence (IPV) are often used interchangeably in the literature [2]. A number of studies from low- and middle-income countries (LMICs) most commonly use the term DV as opposed to the term IPV [3–5]. The use of the term domestic violence is based on the understanding that many women from these countries live within an extended family setting and in some instances, it is the family members who are the perpetrators of DV [4, 6]. The term domestic violence (DV) has been used in this particular review to denote any forms of violence and abuse perpetrated against woman by someone in her family [5, 7].

The significant global burden of DV during pregnancy has been well documented in literature [5, 8]. A recent meta-analysis confirmed a higher proportion of DV during pregnancy in developing countries than developed countries (27.7% versus 13.3%) [9]. DV during pregnancy is particularly alarming in light of its severe negative effects, not only physical but also mental, on a woman, her unborn child, children, and her family [9–11]. It can lead to homicide, suicide, negative health behaviours, preterm birth, repeated miscarriages, poor quality of life (QOL), and developmental delay and restricted growth in children [10, 12]. DV during pregnancy contributes significantly to a number of mental health problems such as anxiety, depression, and post-traumatic stress disorder (PTSD) [13, 14]. DV and mental illness are interconnected and both remain the major source of maternal morbidities [14].

Several interventions aimed at addressing DV and co-morbid health conditions are expanding globally in the last decade. DV screening accompanied by key therapeutic interventions, such as counselling, psychotherapy, and education, has shown some encouraging results [15–17]. However, the generalizability of this finding is problematic as a disproportionately high number of the studies stemmed from high-income countries (HICs), and most of them have not considered DV in the context of pregnancy [16–19].

Pregnancy is identified as a period when there is an increased risk of DV [5, 20]. Yet at the same time, it presents a unique opportunity to identify victims and offers support to them, because of repeated interactions with health care providers (HCPs) from early pregnancy to postpartum [20, 21]. The risk to violence and ability to prevent and cope with it are different for pregnant women compared to non-pregnant population [5, 8]. Jahanfar et al. [22] and Van Parys et al. [23] evaluated DV interventions around the time of pregnancy. A number of DV interventions ranging from a brief, one-session individualized consultation to multiple therapy sessions during pregnancy and some even extending up to postpartum were identified in both reviews [22, 23]. However, due to a limited number of studies and lack of consistency in the outcomes, a meta-analysis could not be performed in either review. Consequently, both reviews failed to provide conclusive recommendations about any one intervention that can be adopted within an antenatal care (ANC) context.

A detailed explanation on the rationale for conducting this review can be found in a previous publication [24]. DV is now considered as a public health priority in several LMICs, and thus, a number of interventions might have been developed in recent years. However, they have not been systematically assessed for comparative efficacy. As violence is a contextual matter, the applicability and efficacy of a particular intervention may vary in different settings [25, 26]. Factors such as financial limitations, inadequate human resources, cultural barriers, social norms, and government policy may impede the ability of LMICs to deliver the interventions that have been found efficacious in HICs [27].

DV interventions are inherently complex, with multiple interacting components. Some researchers suggest that understanding of interventions’ underlying Theory of Change (ToC) may improve their evaluation [28]. ToC is a comprehensive description and illustration of how and why a desired change is expected to happen in a particular context. The development of a ToC is an iterative process and can used various methods including review of existing information, interviews and/or consultation with stakeholders, with the choice of the method being based upon what is locally feasible and acceptable [28]. The use of ToC approach is widespread in the field of public health and many development organizations have evaluated this method as accessible, feasible, and useful [29]. Through this review, we sought to draft a ToC explaining how certain activities can lead to the reduction of DV and improvement of mental health among pregnant women.

### Aims of the review

The main purpose of this review was to obtain a complete representation of interventions available in LMICs for reducing and/or controlling DV among pregnant women and assess their effectiveness. A draft ToC was developed to illustrate the working mechanism of DV interventions in a real-world setting.

## Methods

The Preferred Reporting Items for Systematic Reviews and Meta Analyses (PRISMA) guidelines [30] were used as a framework for this review (Additional file 1). The review protocol was registered in the International Prospective Register of Systematic Reviews (PROSPERO) (CRD42017073938) and published in the Systematic Reviews [24].

### Search strategy

Four key concepts, including “Domestic Violence”, “pregnant”, “LMICs”, and “intervention” were combined and Medical Subject Headings (MeSH), controlled vocabulary, and key words were used to make the search queries exhaustive. Electronic databases such as CINAHL, Web of Science, Scopus, Embase, The Cochrane Library, and psycINFO were systematically searched with no restrictions of the date of publications. The search strategy of Ovid MEDLINE (R) and psycINFO is in Additional file 2. Google scholar, Cochrane Methodology Register, WHO International Clinical Trials Registry Platform (ICTRP) were searched for grey literature. Reference lists of the included studies were cross-checked to identify additional papers of interest. The searches were systematically updated during the writing process, the last update taking place in February, 2018.

### Study selection criteria

The review included studies that (1) included pregnant women of any age; (2) were conducted in LMICs, according to the World Bank income criteria [31]; (3) had evaluated an intervention related to DV; (4) reported at least one outcome of interest; and (5) were published and unpublished articles written in English language. Outcomes of interest included any measures of frequency and/or severity of DV (primary outcome) and changes in mental health outcomes such as QOL, depression, anxiety, stress, self-efficacy, self-esteem, use of safety behaviours or community resources or referral services, and social support (secondary outcomes). Studies that did not separately report outcome data for pregnant women and were conducted in HICs including women from LMICs were excluded. Observational studies were not included.

### Study selection

**Fig. 1:**
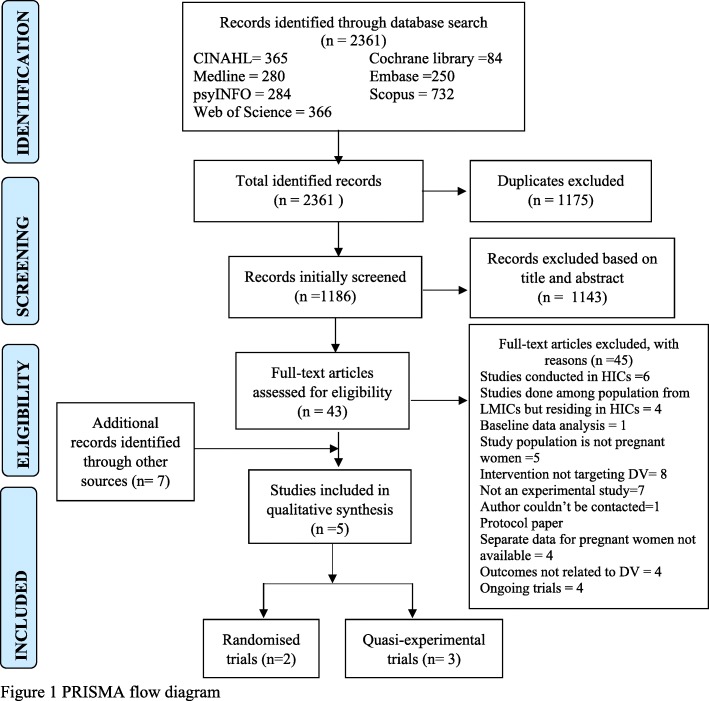
PRISMA flow diagram

Endnote (V.X8) was used to manage and store relevant studies. After removing duplicates, two reviewers independently assessed the eligibility of the studies, and when a difference of opinion occurs, the issue was resolved with consensus involving a third reviewer. The full text of potentially relevant articles was also reviewed independently by two reviewers. Several interactive meetings were conducted among the reviewers to make a final inclusion or exclusion decision of full articles. The reasons for excluding the studies are provided in Additional file 3. Figure 1 presents the flow diagram depicting studies selection process [30].

### Methodological appraisal of study

Classification of risk of bias as recommended by the Cochrane handbook was used to assess the quality of randomized controlled trials (RCTs) [32]. Initially, it was planned to use Risk of Bias in Non-randomized Studies−of Interventions (ROBINS–I) for appraising the risk of bias of non-randomized studies (NRS) [32]. Unfortunately, the selected NRS had used one-group pre- and post-intervention design and it was not feasible to use this tool. Hence, critical appraisal checklist for quasi-experimental studies was used to assess their methodological quality [33] (Additional file 4). Considering the small number of available studies, no studies were excluded based on these assessments of risk of bias.

### Data extraction and analysis

Findings from selected studies were extracted independently by two reviewers on a structured database [24]. As studies were methodologically diverse and reported varied outcome measures and measurement time points, a meta-analysis could not be done. A narrative synthesis of the findings was carried out, which included information on characteristics of the study and study population (such as publication year, study design, sample size, and setting), and description and outcomes of the interventions (e.g., intervention duration, follow-up, content and type of intervention, and outcome measures) [24].

The process of developing a ToC was initiated with an identification of long-term goals of the included interventions. Subsequently, the reviewers worked backwards to develop a pathway of change illustrating the cause-effect relations between the activities and the intended outcomes. Each output was interconnected; in that they influenced and supported the outcomes and facilitated the achievement of desired impacts. Assumptions were articulated to explain the linkages between the activities and outcomes and were supported by existing theories and findings from the included studies.

## Results

### Description of the studies

Systematic searches generated 2368 articles (2361 from electronic databases and 7 from additional sources). Two cluster RCTs, one conducted in Mexico and one in India [34, 35], included both pregnant and non-pregnant women in the study population. Authors of both studies were contacted to provide separate data on pregnant women, however, no additional detail was obtained, and therefore both studies were excluded. One trial which evaluated the effectiveness of an empowerment program for abused pregnant women in India was located in WHO ICTRP (unpublished Sharma, 2013). The author could not be contacted as contact details were not provided. Two trials evaluating the impact of counselling intervention among abused pregnant women, one in Iran (unpublished Sepidah, 2017) and another in Nigeria (unpublished Uchendu, 2017), were identified in ICTRP. As the studies were ongoing, the results were not available. A counselling-based intervention to address DV in antenatal settings has been undertaken in South Africa. The author was contacted to get the update of the study but the results were not yet available [36].

A total of five studies met the inclusion criteria and were included in the review [37–41]. Out of five studies, two were RCTs [37, 40] and three were quasi-experimental (before and after) studies [38, 39, 41]. Among five included studies, three were pilot studies [37, 38, 41]. Identified studies were published in between the years 2010 [37] and 2013 [39–41]. Most (*n* = 4) of the interventions were specifically designed for reducing/controlling DV for pregnant women [37–39, 41], but one was part of a larger, multifaceted intervention primarily targeting human immunodeficiency virus (HIV), and Prevention of Mother to Child Transmission (PMTCT), and DV was one of its component [40]. Three out of five studies were carried out in African countries [39–41], one trial was conducted in Peru [37], and one in India [38]. Important characteristics of selected studies are illustrated in (Additional file 5: Table S1).

DV was assessed by the revised Conflict Tactics Scale [37, 40], Danger Assessment Score [39], and a self-constructed DV risk assessment questions [41]. However, in one study, the tool used to measure DV was not clear [38]. The duration considered for measuring the prevalence of DV varied remarkably across the studies. SF-36 was used to assess the mental health-related QOL [42] and safety behaviour checklist, developed by McFarlane [43], was used to assess the safety behaviours. Use of community resources was assessed by self-constructed questionnaire [37].

### Risk of bias of studies

**Fig. 2:**
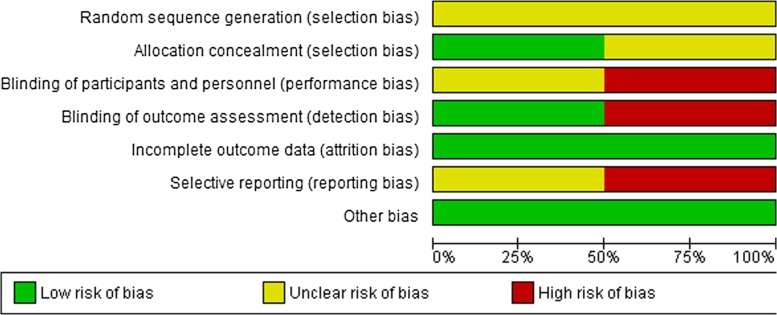
Risk of bias item presented as percentages across all included studies

**Fig. 3:**
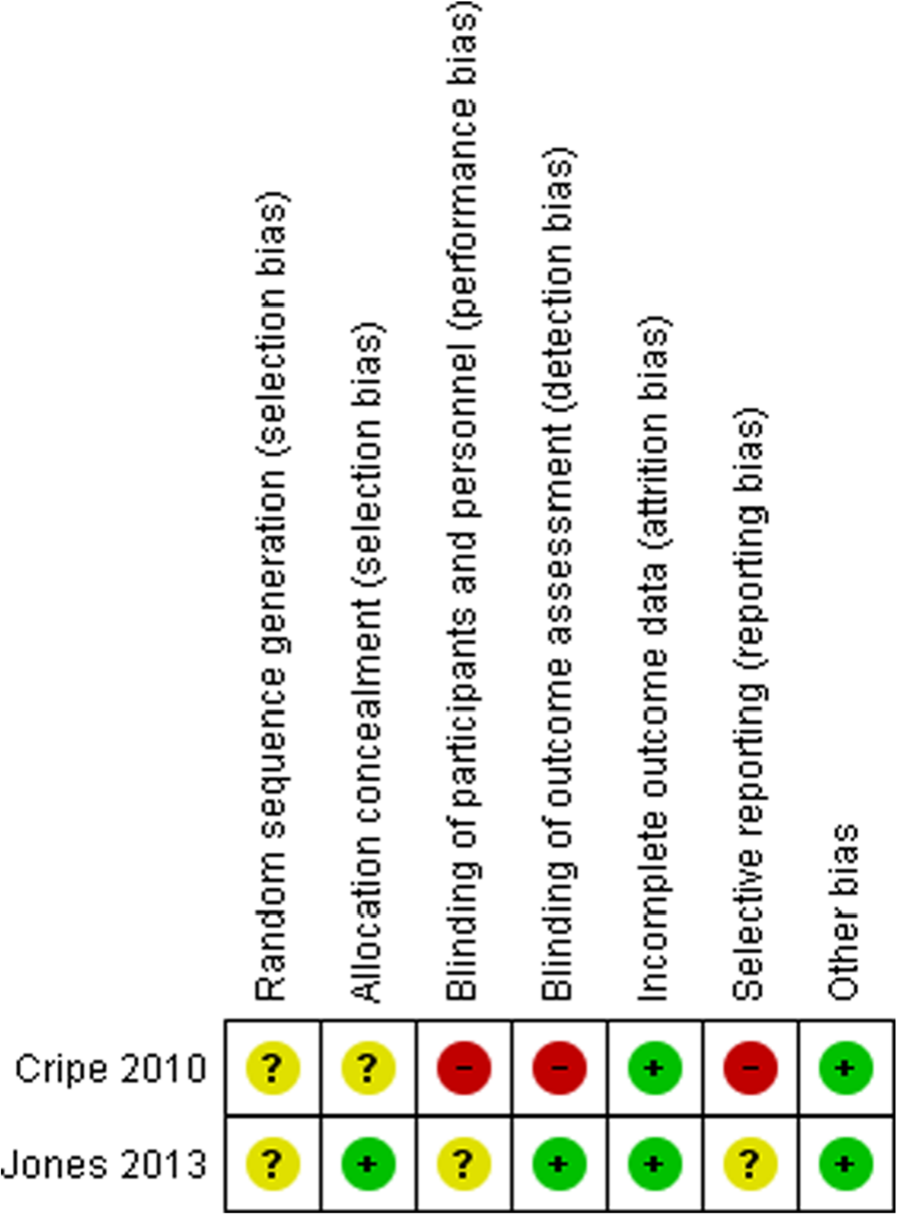
Risk of bias item for each included study

Figures 2 and 3 show a summary of the risk of bias of two randomized studies, which was generated using RevMan 5.0 [44]. In both trials, the method for generating the randomization sequence was not clear [37, 40]. One study had used remote secure randomization service to randomize participants into the two groups [40], and, there was a lack of information on how participants were allocated into the two groups in another study [37]. A study that lacked blinding of participants was considered as having a high risk of bias [37], while in another study, it was unclear if participants were blinded [40]. Post-intervention data collection was performed by different personnel in both trials; therefore, both were judged as low risk of bias [37, 40]. Loss of women to follow-up was very low in both studies [37, 40]. A study by Jones et al. was judged to be unclear because only published study reports were available for the review [40]. Cripe et al. did not report DV-related outcomes post-intervention, thus, rated as at high risk of bias [37]. Both studies appeared to have comparable groups at baseline in terms of participants’ characteristics. No other obvious bias was noted in these studies [37, 40].

Using the critical appraisal checklist [33], quasi-experimental studies were rated as of poor quality due to a number of reasons including small sample size, lack of a control group, and lack of appropriate statistical analysis to draw conclusions (Additional file 4). However, it is important to consider that two out of three studies were pilot studies with primary focus on feasibility and applicability of the interventions [38, 41]. Loss to follow-up was high (47.5%) in a study carried out by Matseke and Peltzer [39]. However, attrition analysis showed that there was no difference in magnitude of DV among women who dropped out of the study and those who did not [39].

### Characteristics of the study population

All of the selected studies included women aged 18 years and above. In total, 1086 participants were recruited in the included studies, with sample sizes ranging from 20 [38] to 478 dyads [40]. Women were socio demographically diverse; the majority of the study population had not completed their higher secondary school education and at the time of the study were unemployed.

### Description of interventions

Most of the interventions were delivered at health care settings and inclined to be relatively brief. The duration of an intervention session ranged from a one-time session lasting for 20 min [39] to four weekly sessions, each lasting for 90–120 min [40]. The duration of follow-up period also varied considerably between the different studies; follow-up ranged from immediately [38] to 3 months post-intervention [39]. Turan et al. reported that for safety reasons, they did not follow-up the women who accepted referrals to DV support services [41]. Interventions were compared with either usual care or standard care. Standard care usually involved regular ANC care or health care.

### Narrative theory of change (ToC) for a DV intervention

**Fig. 4:**
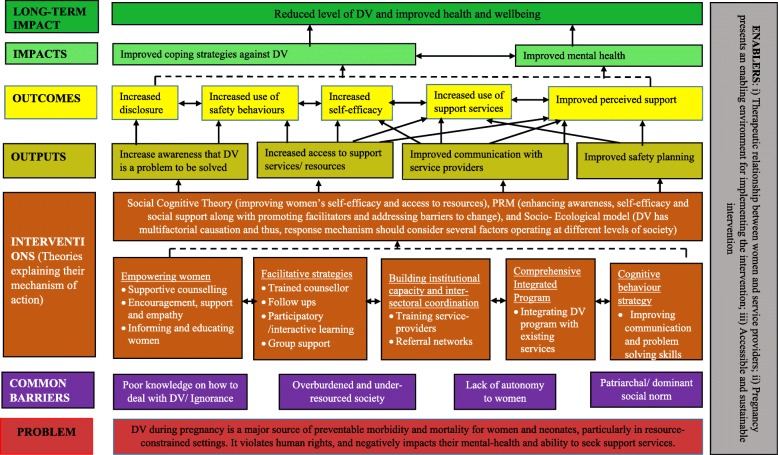
A theory of change for interventions addressing DV among pregnant women in LMICs

This section briefly describes the individual component of a ToC. A schematic representation of the ToC is illustrated in Fig. 4.

#### Context

The foundation of the ToC begins with a core problem that DV during pregnancy contributes to a larger proportion of maternal and neonatal morbidities and mortalities in LMICs. Additionally, it is accepted that there is a lack of critical awareness about gender and rights among women and that DV is frequently normalized and generally accepted in family relationships. The continuing dominant patriarchal norms reinforce the submissive role of women [4]. Moreover, there is significant deficit of resources and services to deal with DV effectively.

#### Activities

A number of intervention strategies or activities contribute directly or indirectly to overcome these barriers, which are described below in brief.

##### Screening and empowerment of pregnant women

The core component of all interventions was supportive counselling and mentoring to women [37, 39, 41]. Most of the studies had counselled women individually, while two studies had conducted group counselling sessions [38, 40]. With the counselling and support, these interventions aimed to aware and empower women [37–41].

##### Facilitative strategies

Frequent check-ins with women help to underpin and reinforce their learning as well as support them to access support services if needed [40]. Interactive discussions with trained counsellors help women clarify doubts and gain a better understanding of existing response mechanisms [37–40].

##### Capacity building of health and non-health workers

HCPs or social workers were trained on a number of topics, such as screening DV, addressing physical and emotional needs of victims, referring abused women to support services, and adhering to ethical principles [37–41].

##### Comprehensive integrated program

DV programmes were comprehensive and included information on DV, developing communication skills, relationship skills training, and safety planning and supported referrals [37–41]. Participants were provided with a referral list of DV support services [37, 39, 41] and were encouraged and assisted in seeking help at times of need [39, 41]. In a study from South Africa, DV intervention was integrated with HIV PMTCT program [40]; while in other studies, interventions were delivered during women’s ANC visits [37, 38].

##### Developing cognitive behavioural skill

A study by Jones et al. emphasized on cognitive behavioural therapy (CBT), enhancing choice-making and problem-solving to improve communication, sexual negotiation, and conflict resolution [39, 40].

#### Assumptions

Assumptions explain the possible mechanisms of one outcome leading to another. The intervention components can on its own or in combination influence these outcomes, and the outcome pathway is depicted in a logical relationship.

- Awareness of physical, mental, and social consequences of DV motivates women in taking action against it.
- Active participation and safe interaction within the group supports women to disclose their experiences of abuse and access support services.
- Trained service providers can address the physical and emotional needs of the victims and assist them in accessing referral networks.
- Integrating DV intervention into existing health services improves its acceptance and accessibility.
- CBT improves communication and problem-solving skills. It helps person to get rid of negative thoughts and cultivate positive thoughts.
- Effective coping against DV requires improved self-efficacy and increased use of safety behaviours and support services. When women feel that they are continually supported in their social environment, they may feel more positive about themselves and therefore be resilient to setbacks.
- Empowerment acts as means as well as ends of DV intervention. Self-awareness enhances self-care which in turn improves women’s self-esteem. Women develop competency in adopting effective response mechanisms against DV and emotional stress.
- With this level of awareness and empowerment, the victimisation and re-victimisation of women can be reduced, and health and wellbeing can be enhanced.

#### Evidence

Evidence from the included studies and existing theories is used to support the abovementioned assumptions. Disclosure of abuse and corresponding validation of feelings helped women understand that DV is common and help is available [37]. Training to service providers addressed gaps in both their knowledge and skills and prepared them to deal with disclosure of abuse effectively [37, 41]. Cripe et al. unearthed a trend towards an improved QOL (mental), and safety- and help-seeking behaviours in women receiving supportive counselling [37]. There was a significant reduction in mean danger assessment score after 3 month of the intervention (6.0 vs 2.8) [39]. Jones et al. reported a decrease in at least one act of violence among women randomized to counselling (*p* < 0.001) compared with those receiving usual care [40]. Krishnan et al. reported an improvement in relationships of pregnant women with their mothers-in-law post-intervention [38]. In a 5-month period, a total of 134 pregnant women were screened for presence of DV and 53% of those screened positive (*n* = 49) accepted referrals to local support services [41].

The implicit theory underlying most of the interventions was that the supportive counselling along with guided referrals resulted in increased critical awareness and assisted women in goal-setting and decision-making. This aligns with the social cognitive theory which highlights that the improvements in self-awareness, empowerment, and self-efficacy are critical elements for behaviour modification [45]. The causes of DV are complex and contextual [46]. The socio-ecological model highlights the significant influences of society and community on the occurrence of DV during pregnancy [7]. Addressing only one single risk factor might not be successful, as other factors can act as barriers to desired changes [37, 40]. Hence, a context-specific intervention focussing on possible interplay of multiple factors operating at different levels are more likely to exhibit positive effects on reducing DV and managing its health consequences. Increased awareness coupled with enhanced support and access to resources is often considered instrumental in behaviour change. Psychosocial readiness model (PRM) supports this assumption, which considers that when all three internal factors: awareness, self-efficacy, and perceived support, are intact, women feel the readiness to change [47]. However, an impediment in any one factor and/or the presence of barriers in external environment leads to a resistance to change. Interventions needs to be comprehensive enough to bolster internal factors and manage external influencers [47]. PRM has been widely adopted in recent DV research [48, 49] as it takes into account the changeable nature of women’s actions and desires, and acknowledges improvement in QOL and help-seeking behaviours as desirable actions [47].

### Enablers

- Service providers need to be non-judgemental, empathetic, and supportive. Similarly, women need to develop strong relationship of trust, honesty, and openness with service providers for the successful delivery of an intervention (internal enablers).
- Pregnancy is a period when women are in regular interaction with HCPs and are motivated to change or alter their situation (internal enablers).
- Long-term success depends on the uninterrupted availability of the intervention and ongoing organizational commitment (external enablers).

## Discussion

This review sheds light on both the narrow and inconclusive evidence with regards to the effectiveness of DV interventions among pregnant women, which is in accordance with the findings of previous reviews [22, 23].

Violence is a contextual matter and is inherently associated with several ethical and safety challenges. This has led to considerable variation within the studies; namely, measurement tools, research settings, sample sizes, content of intervention, and duration of follow-up. Rather than telling a woman what she must or should do, all interventions were based around the concept of empowerment; assisting the woman to disclose her experiences of abuse, identifying the best available resources as well as helping her to find a potential solution that would best fit with her situation. Hence, it can be argued that the interventions included in this review were pragmatic, seeking to provide tailored services to meet the women’s individual needs and circumstances. Similar intervention components were noted in other studies carried out in HICs [49–51].

Designing a feasible intervention that is likely to work in the constraints of the context and available resources is challenging. The ToC diagram has included the factors that possibly act as barriers in the successful implementation of interventions addressing DV in LMICs and highlighted the need of creating a supportive environment to address those barriers effectively. However, these barriers are not exhaustive, and for an intervention to be effective, it must respond to context-specific factors [52]. The ToC may improve the initial design and potential effectiveness of the intervention by explicitly demonstrating multiple pathways towards the intended outcomes. However, in reality, the processes are not always linear; rather they are complicated, multi-directional, and highly context-specific. Hence, rather than considering it as a prescriptive map, further studies need to use and expand this knowledge to identify knowledge gaps and generate research questions. Our approach of developing ToC has been relatively simplistic with the potential to be further developed. Further rigorous discussion with different stakeholders is recommended to make the ToC more robust. Empirical testing of the assumptions in future research may help reduce implementation failure by identifying weak associations in the casual pathway and guiding the revision of intervention.

This review indicated that the interventions addressing DV are just beginning to emerge in developing countries. A growing number of existing HIV and reproductive health programmes are now beginning to integrate DV into their activities. Literature has shown that DV often overlaps with the HIV epidemic and these linkages have prompted the testing and scaling of integrated interventions, mostly in an epidemic region like Africa [53]. DV interventions delivered in antenatal settings were found to be effective in studies conducted in HICs [50, 54]. Even though this review was unable to provide enough evidence to confirm the success of such integration within LMICs, delivering an integrated DV intervention during a woman’s antenatal visit is still recommended. Indeed, designing a multifaceted intervention may be preferable to standalone intervention as they can be more cost-effective and acceptable by the victims [2].

Despite the tremendous effect of victimization and ongoing abuse on emotional wellbeing of victims, collaborative models for addressing these issues to date have been slow to develop [14, 22]. Women with poor mental health may find it inherently difficult to adhere to the recommended safety plan and utilize the resources effectively [55]. In this review, only one study reported on the insignificant effect of DV intervention on a woman’s mental health. Therefore, it was not possible to draw a conclusion on what intervention contributes to the improvement in the woman’s mental health and wellbeing. Nevertheless, it can be assumed that QOL may be ensured if a woman feels safe at home and confident to access resources at times of need. Self-efficacy and social support have shown a positive effect in improving psychological distress symptoms [54, 56]. WHO clinical and policy guidelines, 2013, advocates that the first line response for victims of DV should include listening, inquiring about needs, validating women’s experiences, enhancing safety, and ensuring support (LIVES) in health settings [2]. Hence, it is necessary to integrate mental health component in DV intervention which can assist women in restoring their mental strength and wellbeing while making appropriate life decisions.

Despite being the first systematic review evaluating DV interventions among pregnant women in LMICs, it must be acknowledged that this review has certain limitations. For example, the heterogeneity across the intervention studies restricted the use of meta-analysis. Several reasons can be inferred for the insignificant results obtained in the studies. For instance, some interventions might have been effective but due to some methodological limitations, they might have failed to reach statistical significance level [37]. Additionally, offering a referral card to the control group can itself act as an intervention [37], as it might sensitize the victims to seek additional information and use resources when needed. Studies were unable to maintain the blinding of research personnel and participants due to the typical nature of intervention which might have introduced contamination between the groups. A language bias has to be considered as only articles published in English were included in this review. However, as there is increased tendency and popularity of publishing in English journal, this restriction might be less significant. Use of subjective judgement throughout the review process might also be responsible for variability across the evaluation. However, every effort was made to minimize this bias in the review, such as independent assessment and extraction of data by two reviewers, and involvement of a third reviewer in the case of difference of opinion.

This review does provide some important implications for practice and future research. The ToC can serve as a framework to inform future research. Since, ToC development is a continual process, future research is recommended to further refine the ToC. Future research should utilize the rigorous methodology, such as RCTs, to analyze the efficacy, usability, and sustainability of a DV intervention. This ToC could be relevant to practice in different ways: (1) it could be used as a guide in developing DV programmes, (2) it could be used to interpret research and review, and (3) it could be used by policy-makers and implementing organizations for informed decision-making.

## Conclusion

The substantial heterogeneity of interventions and mixed findings prevented us from drawing any firm conclusions, although some important recommendations and implications for future research can be made from this review. Though the paucity of effective interventions addressing DV among pregnant women in LMICs was evident, available studies supported the use of counselling-based intervention involving safety planning and guided referrals. The development of a ToC was critical in understanding the context in which DV interventions worked to produce the desired outputs. This can provide valuable input for design and evaluation of DV program in the future.

## Additional files

Additional file 1: PRISMA 2009 Checklist. (DOC 64 kb)

Additional file 2: Medline Search Strategy. (DOCX 23 kb)

Additional file 3: List of studies excluded from the review including reasons for their exclusion. (DOCX 31 kb)

Additional file 4: Quality appraisal of before and after studies. (DOCX 13 kb)

Additional file 5: **Table S1.**Overview of studies in the review. (DOCX 49 kb)

## Abbreviations

CBT: Cognitive behavioural therapy
DFV: Domestic and family violence
DV: Domestic violence
GBV: Gender-based violence
HCPs: Health care providers
HICs: High-income countries
HIV: Human immunodeficiency virus
IPV: Intimate partner violence
LMICs: Low- and middle-income countries
MeSH: Medical Subject Headings
NRS: Non-randomized studies
PMTCT: Prevention of mother to child transmission
PRISMA: Preferred Reporting Items for Systematic Review and Meta-analysis
PRM: Psychosocial readiness model
PROSPERO: International Prospective Register of Systematic Reviews
PTSD: Post-traumatic stress disorder
RCTs: Randomized controlled trials
ROBINS−I: Risk of bias in non-randomized studies−of interventions
ToC: Theory of change
VAW: Violence against women
WHO: World Health Organization
