# Supplementary material for: Interventions for reducing and/or controlling domestic violence among pregnant women in low- and middle-income countries: a systematic review
Source: Syst Rev. 2019 Apr 2;8:79. doi: 10.1186/s13643-019-0998-4 (PMC6889323; doi:10.1186/s13643-019-0998-4)
Supplement: Supplementary file 3 — List of studies excluded from the review including reasons for their exclusion. (DOCX 31 kb) [file 13643_2019_998_MOESM3_ESM.docx]

**List of studies excluded from the review including reasons for their exclusion**

| S.N. | Study | Reason for exclusion |
| --- | --- | --- |
| 1. | Bryant et al., 2017 | The study had evaluated the effectiveness of a brief behavioural intervention on women with a history of abuse. The principal investigator was contacted to provide separate information for pregnant women via email, however there was no separate data for pregnant women. |
| 2. | Gupta et al., 2017 | The study evaluated the effectiveness of nurse-led intervention in addressing DV among abused women. It has included both pregnant and non-pregnant women. Author was contacted for providing separate data for pregnant women. However, there was no separate data and thus the study was excluded. |
| 3. | Raj et al., 2016 | The study has included both pregnant and non-pregnant women. Author was contacted for providing separate data for pregnant women. However, there was no separate data and thus the study was excluded. |
| 4. | Peltzer et al., 2016 | The study reported cross-sectional analysis of baseline information of participants included in a cluster randomized trial implemented in South Africa. |
| 5. | Dugravier et al., 2013 | The study had evaluated the impact of multifocal perinatal home-visiting intervention using psychologist on postnatal depression. The intervention did not have specific DV related component and had not measured DV as well. |
| 6. | Frith et al., 2017 | The study evaluated the effect of breast counselling intervention on mitigating the negative effect of DV on exclusive breast feeding. The intervention was not related to addressing the DV. |
| 7. | Masters et al., 2016 | The intervention was primarily targeting the promotion of partner testing and couple testing and occurrence of DV was measured as an adverse event of the tested intervention only. |
| 8. | Mohlala et al., 2016 | The intervention basically aimed to invite male partner to attend couple voluntary counselling and testing and occurrence of DV was measured as an adverse event of the tested intervention only. |
| 9. | Sikkema et al., 2010 | This intervention study evaluated the impact of HIV prevention strategies integrated with issues of gender and power imbalance. . The principal investigator was contacted to provide separate information for pregnant women via email. The PI responded that as the study was not specific to pregnancy, there was no separate data. |
| 10. | Orne-Gliemann et al., 2013 | Couple oriented post-test HIV counselling was provided to the intervention group and DV was measured as adverse effect of the intervention only. |
| 11. | Osoti et al., 2014 | Home based vs clinic based HIV counselling was compared between two groups of women and DV was measured as adverse effect of the intervention only. |
| 12. | Rosenberg et al., 2015 | The intervention did not primarily addressed the domestic violence among pregnant women. |
| 13 | Ziaei et al., 2016 | The study presented the analysis of baseline information of participants who participated in a nutritional trial named MINIMat randomized trial. |
| 14 | El-Khorazaty et al., 2007 | All these articles belonged to the study named DC Healthy Outcomes of Pregnancy Expectations (DC-HOPE). Though this study has evaluated the effectiveness of DV related intervention, it has recruited African-American women residing in Washington DC. This does not match with our inclusion criteria of the study and thus these studies were excluded. |
| 15. | Katz et al., 2008 |  |
| 16. | Mohandes et al., 2005 |  |
| 17. | Subramanian et al., 2012 |  |
| 18. | Adjiwanou & LeGrand., 2014 | This study is not an interventional study and has analysed the Demographic and Health Survey Data of four sub-Saharan African Countries. |
| 19. | Davis et al., 2017 | This article included information about the participants recruited in the control group of a randomized controlled trial. |
| 20. | Fisher et al., 2015 | This is a prospective cohort study without any intervention or manipulation. |
| 21. | Jewkes et al., 2006 | Baseline information of participants participated in a randomized trial named Stepping stones was done to explore the relationship between HIV infection and IPV among young rural women in South Africa. The intervention was primarily for reducing HIV infection and not targeted for pregnant women. |
| 22. | Koen et al., 2014 | The study reported data from The Drakenstein Child Lung Health Study (DCLHS) which aimed to investigate the epidemiology and etiology of childhood respiratory illness and the determinants of child health in a low socio-economic area in South Africa. |
| 23. | Nunes et al., 2010 | It is a cohort study conducted among pregnant women attending 18 primary care units in Brazil with first assessment at 16-36 weeks of pregnancy and follow-up included immediate and at 4-5 month postpartum assessment. |
| 24. | Suarez Ordonez et al., 2015 | It included the review of literature to outline the importance of screening for behavioural problems in pregnant women and offer a comprehensive and dynamic behavioural health screening model that could be implemented in Argentina. |
| 25. | Ali et al., 2009 | The study not related to domestic violence and did not included DV outcome. |
| 26. | Ammermam et al., 2016 | The study evaluated the effectiveness of Cognitive Behavioural Therapy in depressed mothers and has not evaluated any component related to DV. |
| 27. | Grote et al., 2012 | The study evaluated the impact of childhood trauma on the outcomes of a perinatal depression trial. |
| 28. | Jewkes et al., 2008 | This randomized trial implemented in South Africa has evaluated the effect of intervention aimed to build knowledge, risk awareness and communication skills. The main outcome measures was incidence of HIV and HSV-2 and included school going women of age 15-26 years. |
| 29. | Krishnan et al., 2012 | The article is about the protocol of Dil Mil trial implemented in India and did not include any study findings. |
| 30. | Dauber et al., 2017 | The study was conducted in high income settings. |
| 31. | McFarlane et al., 1998 | The study was conducted in high income settings. |
| 32. | McFarlane et al., 1999 | The study was conducted in high income settings. |
| 33. | Nicolaidis et al., 2013 | The study was conducted in high-income settings (Portland, Oregan, USA). |
| 34. | Olds et al., 2004 | The study was conducted in high income settings (USA). |
| 35. | Zlotnick et al., 2011 | The study was conducted in high income settings (USA). |
| 36. | Krishnan et al., 2016 | The study population were not pregnant women. |
| 37. | Patel et al., 2017 | The study population were not pregnant women. |
| 38. | Tiwari et al., 2010 | The study population were not pregnant women and the study recruited Chinese women residing in HIC i.e. Hong kong. |
| 39. | Sarnquist et al., 2014 | The study population is adolescent girls. |
| 40. | Bahadir-Yilmaz et al., 2018 | Author was contacted requesting separate data for pregnant women, however, there was no reply. |

Characteristics of ongoing studies

| Study | Characteristics | Details |
| --- | --- | --- |
| 1. Krishnan, 2012 | Trial name or title | The Dil Mil Trial |
|  | Country | India |
|  | Study design | RCT using a parallel comparison. |
|  | Participants | Young married pregnant women (18 to 30 years old) with a history of domestic violence in the first or second trimester of pregnancy (daughter-in-laws (DILs) and their mother-in-laws' (MILs). |
|  | Interventions | Intervention consists of 2 half-day group sessions with DILs, 5 half-day group sessions with MILs, and 1 joint half-day session with DILs and MILs. |
|  | Outcomes | Domestic violence incidence, related health outcomes (perceived quality of life, psychosocial status, and maternal and infant health), knowledge of links between GBV and health, gender-equitable attitudes, relationship with DILs (communication social support, support of freedom of movement), reduction in GBV perpetration, resistance to GBV inflicted by son, knowledge of safety and the links between GBV and health, gender-equitable attitudes, decision-making skills, freedom of movement, resistance to GBV by husband. |
|  | Study details | Starting and ending date of study were unclear |
|  | Notes | The principal investigator of the study was contacted via email but there was no response. The findings from the pilot study of the trial has been included in the review. |
| 1. Sharma, 2013 | Trial name or title | Impact of women’s empowerment program for abused pregnant women |
|  | Country | India |
|  | Study design | Randomized trial with parallel assignment |
|  | Participants | 18-35 years pregnant women with a history of violence |
|  | Interventions | Intervention Group: The hour intervention, delivered over an 11-week period, consisted of an empowerment and additional components adapted from Freedom program run to support domestic abused women.  Control group: Standard care which included routine check ups and care provided by health care professionals |
|  | Outcomes | Quality of life, Postnatal depression, domestic violence and safety behaviour |
|  | Study details | Started on January 2013 and completed on August 2013 |
|  | Remarks | The principal investigator could not be contacted as there was no contact details. |
| 1. Moeini, 2017 | Trial name or title | The Effect of Preventive Intimate Partner Violence Interventions for Mental Health Promotion among Pregnant Women in Marginal Areas |
|  |  | Iran |
|  | Study design | Non-blinded randomised trial with parallel assignment |
|  | Participants | 18 years and older pregnant women, married for at least 3 years and having at least one child from current marriage, living permanently in the study area and have a spouse in life. |
|  | Interventions | Behavioural intervention with education component. |
|  | Outcomes | Domestic violence and mental health |
|  | Study details | First enrolment 2017-11-12 and expected recruitment end date 2010-03-19. |
|  | Remarks | Recruitment of participants is ongoing and no results of the intervention available. |
| 1. Uchendu, 2017 | Trial name or title | The effect of a counseling Intervention on Risk of IPV in Pregnant women in Nigeria |
|  | Country | Nigeria |
|  | Study design | Parallel group randomized study |
|  | Participants | ANC attendees who are at least 18 years of age, with gestational age 24 weeks or less. |
|  | Interventions | IPV screening, risk assessment and counselling |
|  | Outcomes | DV |
|  | Study details | Trial start date 2017-12-29 and anticipated date of last follow up 2018-03-29 |
|  | Remarks | Email sent requesting further information and the author responded that the data is being collected and the result is not yet available. |
| 1. Sepidah, 2017 | Trial name or title | The effect of solution focused counseling on violence rate and quality of life of pregnant women at risk of domestic violence |
|  | Country | Iran |
|  | Study design | Randomized trial |
|  | Participants | Pregnant women aged 18 years and above with gestational age of 27 weeks or less, with a history of violence, living with husband for at least one year |
|  | Interventions | Individual solution focused counselling during six sessions of 90 minutes and all the counselling classes will be held by the researcher. In order to achieve higher efficacy, all participants will receive positive key messages at the end of each session. At the end of each two counselling session, main points of each session will be presented. The control group will receive no counselling. |
|  | Outcomes | Domestic violence, Quality of life |
|  | Study details | Recruitment complete |
|  | Remarks | The trial is ongoing and results are not yet available. |
| 1. Pallito, 2016 | Trial name or title | ‘Safe and Sound’ intervention. Addressing violence against pregnant women in antenatal care: testing an intervention in South Africa |
|  | Country | Johannesburg, South Africa |
|  | Study design | Two-arm randomized controlled trial |
|  | Participants | Women who are at least 18 years old and less than 33 weeks gestation, have a history of DV in past 12 months. |
|  | Interventions | Two sessions of empowerment based counselling was provided by trained nurse. The women were provided with the information about the DV, safety measures and they were assisted to seek available support services to cope with DV effectively. |
|  | Outcomes | DV, mental health, safety planning, community resource use and self-efficacy |
|  | Study details | Study is completed |
|  | Remarks | Author was contacted to provide the results, but the results were not available. |

**References of excluded studies**

1. Bryant RA, Schafer A, Dawson KS, Anjuri D, Mulili C, Ndogoni L, Koyiet P, Sijbrandij M, Ulate J, Shehadeh MH et al. Effectiveness of a brief behavioural intervention on psychological distress among women with a history of gender-based violence in urban Kenya: A randomised clinical trial. Plos Medicine. 2017;14(8).
2. Gupta J, Falb KL, Ponta O, Xuan Z, Campos PA, Gomez AA, Valades J, Cariño G, Olavarrieta CD. A nurse-delivered, clinic-based intervention to address intimate partner violence among low-income women in Mexico City: findings from a cluster randomized controlled trial. BMC Medicine. 2017;15(1):128.
3. Raj A, Ghule M, Ritter J, Battala M, Gajanan V, Nair S, Dasgupta A, Silverman JG, Balaiah D, Saggurti N. Cluster randomized controlled trial evaluation of a gender equity and family planning intervention for married men and couples in rural India. PloS One. 2016; 11.
4. Peltzer K, Rodriguez VJ, Jones D. Prevalence of prenatal depression and associated factors among HIV-positive women in primary care in Mpumalanga province, South Africa. Sahara J. 2016;13(1):60-67.
5. Dugravier R, Tubach F, Saias T, Guedeney N, Pasquet B, Purper-Ouakil D, et al. Impact of a Manualized Multifocal Perinatal Home-Visiting Program Using Psychologists on Postnatal Depression: The CAPEDP Randomized Controlled Trial. Plos One. 2013;8(8).
6. Frith AL, Ziaei S, Naved RT, Khan AI, Kabir I, Ekstrom EC. Breast-feeding counselling mitigates the negative association of domestic violence on exclusive breast-feeding duration in rural Bangladesh. The MINIMat randomized trial. Public Health Nutrition. 2017;20(15):2810-18.
7. Masters SH, Agot K, Obonyo B, Mavedzenge SN, Maman S, Thirumurthy H. Promoting Partner Testing and Couples Testing through Secondary Distribution of HIV Self-Tests: A Randomized Clinical Trial. Plos Medicine. 2016;13(11).
8. Mohlala BKF, Boily MC, Gregson S. The forgotten half of the equation: Randomized controlled trial of a male invitation to attend couple voluntary counselling and testing. AIDS. 2011;25(12):1535-41.
9. Sikkema KJ, Neufeld SA, Hansen NB, Mohlahlane R, Van Rensburg MJ, Watt MH, Fox AM, Crewe M. Integrating HIV Prevention into Services for Abused Women in South Africa. Aids and Behavior. 2010;14(2):431-39.
10. Orne-Gliemann J, Balestre E, Tchendjou P, Miric M, Darak S, Butsashvili M, et al: Increasing HIV testing among male partners. AIDS. 2013;27:1167-1177.
11. Osoti AO, John-Stewart G, Kiarie J, Richardson B, Kinuthia J, Krakowiak D, Farquhar C. Home visits during pregnancy enhance male partner HIV counselling and testing in Kenya: a randomized clinical trial. AIDS. 2014;28(1):95-103.
12. Rosenberg NE, Mtande TK, Saidi F, Stanley C, Jere E, Paile L, et al. Recruiting male partners for couple HIV testing and counselling in Malawi's option B+ programme: an unblinded randomised controlled trial. The lancet HIV. 2015;2:e483-491.
13. Ziaei S, Frith AL, Ekstrom EC, Naved RT. Experiencing lifetime domestic violence: Associations with mental health and stress among pregnant women in Rural Bangladesh: The MINIMat randomized trial. PLoS One. 2016;11(12).
14. El-Khorazaty MN, Johnson AA, Kiely M, El-Mohandes AAE, Subramanian S, Laryea HA, et al. Recruitment and retention of low-income minority women in a behavioral intervention to reduce smoking, depression, and intimate partner violence during pregnancy. BMC Public Health. 2007;7.
15. Katz KS, Blake SM, Milligan RA, Sharps PW, White DB, Rodan MF, et al. The design, implementation and acceptability of an integrated intervention to address multiple behavioral and psychosocial risk factors among pregnant African American women. BMC Pregnancy and Childbirth. 2008;8.
16. Mohandes A, Kiely M, Khorazaty N, Gantz M, Blake S, Subramanian S. Reduction of intimate partner violence in pregnancy: the effect of an integrated intervention in an African-American low income population. Pediatric Academic Societies Annual Meeting; 2005 may 14-17; Washington DC, USA. 2005.
17. Subramanian S, Katz KS, Rodan M, Gantz MG, El-Khorazaty NM, Johnson A, et al. An integrated randomized intervention to reduce behavioral and psychosocial risks: Pregnancy and neonatal outcomes. Maternal and Child Health Journal. 2012;16(3):545-54.
18. Adjiwanou V, LeGrand T. Gender inequality and the use of maternal healthcare services in rural sub-Saharan Africa. Health & Place. 2014;29:67-78.
19. Davis EC, Rotheram-Borus MJ, Weichle TW, Rezai R, Tomlinson M. Patterns of alcohol abuse, depression, and intimate partner violence among township mothers in South Africa over 5 years. AIDS and Behavior. 2017;21:174-182.
20. Fisher J, Tran T, Nguyen T, Nguyen H, Tran T. Common mental disorders among women, social circumstances and toddler growth in rural Vietnam: A population-based prospective study. Child: Care, Health and Development. 2015;41(6):843-52.
21. Jewkes R, Dunkle K, Nduna M, Levin J, Jama N, Khuzwayo N, et al. Factors associated with HIV sero-status in young rural South African women: connections between intimate partner violence and HIV. International Journal of Epidemiology. 2006;35:1461-68.
22. Koen N, Wyatt GE, Williams JK, Zhang M, Myer L, Zar HJ, et al. Intimate partner violence: Associations with low infant birthweight in a South African birth cohort. Metabolic Brain Disease. 2014;29(2):281-99.
23. Nunes MA, Ferri CP, Manzolli P, Soares RM, Drehmer M, Buss C, et al. Nutrition, mental health and violence: From pregnancy to postpartum Cohort of women attending primary care units in Southern Brazil-ECCAGE study. BMC Psychiatry. 2010;10:66.
24. Suarez Ordoñez RM, Cesolari J, Ofelia C, Villavicencio I, Jones HE. Behavioral health screening and intervention for women in Argentina: A preliminary model for the childbearing years. International Journal of Women's Health. 2015;7:635-643.
25. Ali NS, Ali BS, Azam IS. Postpartum anxiety and depression in peri-urban communities of Karachi, Pakistan: a quasi-experimental study. BMC Public Health. 2009;9.
26. Ammerman RT, Peugh JL, Teeters AR, Putnam FW, Van Ginkel JB. Child Maltreatment History and Response to CBT Treatment in Depressed Mothers Participating in Home Visiting. Journal of Interpersonal Violence. 2016;31(5):774-91.
27. Grote NK, Spieker SJ, Lohr MJ, Geibel SL, Swartz HA, Frank E, et al. Impact of childhood trauma on the outcomes of a perinatal depression trial. Depression and Anxiety. 2012; 29(7):563-73.
28. Jewkes R, Nduna M, Levin J, Jama N, Dunkle K, Puren A, et al. Impact of Stepping Stones on incidence of HIV and HSV-2 and sexual behaviour in rural South Africa: cluster randomised controlled trial. British Medical Journal. 2008;337(7666).
29. Krishnan S, Subbiah K, Chandra P, Srinivasan K. Minimizing risks and monitoring safety of an antenatal care intervention to mitigate domestic violence among young Indian women: The Dil Mil trial. BMC Public Health. 2012;12(1).
30. Dauber S, John T, Hogue A, Nugent J, Hernandez G. Development and implementation of a screen-and-refer approach to addressing maternal depression, substance use, and intimate partner violence in home visiting clients. Children and Youth Services Review. 2017; 81:157-67.
31. McFarlane J, Parker B, Soeken K, Silva C, Reel S. Safety behaviors of abused women after an intervention during pregnancy. JOGNN. 1998;27(1):64-69.
32. McFarlane J, Wiist W, Soeken K: Use of counseling by abused pregnant Hispanic women. Journal of Women's Health. 1999;8(4):541-546.
33. Nicolaidis C, Mejia A, Perez M, Alvarado A, Celaya-Alston R, Quintero Y, et al. Proyecto Interconexiones: A Pilot Test of a Community-Based Depression Care Program for Latina Violence Survivors. Progress in Community Health Partnerships. Research Education and Action. 2013;7(4):395-401.
34. Olds DL, Robinson J, Pettitt L, Luckey DW, Holmberg J, Ng RK, et al. Effects of home visits by paraprofessionals and by nurses: age 4 follow-up results of a randomized trial. Pediatrics. 2004;114:1560-1568.
35. Zlotnick C, Capezza NM, Parker D. An interpersonally based intervention for low-income pregnant women with intimate partner violence: a pilot study. Archives of Women’s Mental Health. 2011;14(1):55-65.
36. Krishnan S, Gambhir S, Luecke E, Jagannathan L. Impact of a workplace intervention on attitudes and practices related to gender equity in Bengaluru, India. Global Public Health. 2016;11(9):1169-84.
37. Patel V, Weobong B, Weiss HA, Anand A, Bhat B, Katti B, et al. The Healthy Activity Program (HAP), a lay counsellor-delivered brief psychological treatment for severe depression, in primary care in India: a randomised controlled trial. The Lancet. 2017; 389(10065):176-185.
38. Tiwari A, Fong DYT, Yuen KH, Yuk H, Pang P, Humphreys J, et al. Effect of an Advocacy Intervention on Mental Health in Chinese Women Survivors of Intimate Partner Violence A Randomized Controlled Trial. JAMA. 2010;304(5):536-543.
39. Sarnquist C, Omondi B, Sinclair J, Gitau C, Paiva L, Mulinge M, et al. Rape prevention through empowerment of adolescent girls. Pediatrics. 2014; 133: e1226-1232.
40. Bahadir-Yilmaz E, Oz F. The Effectiveness of Empowerment Program on Increasing Self-Esteem, Learned Resourcefulness, and Coping Ways in Women Exposed to Domestic Violence. Issues in Mental Health Nursing. 2018;39(2):135-141.

**References of ongoing studies**

1. Krishnan S, Subbiah K, Chandra P, Srinivasan K. Minimizing risks and monitoring safety of an antenatal care intervention to mitigate domestic violence among young Indian women. The Dil Mil trial. BMC Public Health. 2012;12:943.
2. Sharma N. Impact of women's empowerment program for abused pregnant women: A randomized controlled trial.  ClinicalTrials.gov [https://clinicaltrials.gov/ct2/show/ NCT01933555](https://clinicaltrials.gov/ct2/show/%20NCT01933555). Accessed 7 March 2018.
3. Noeini B, Rastegar EK. The Effect of Preventive Intimate Partner Violence Interventions for Mental Health Promotion among Pregnant Women in Marginal Areas. International Clinical Trial Registry Platform, WHO. 2017. [http://apps.who.int/trialsearch/ Trial2.aspx?TrialID](http://apps.who.int/trialsearch/%20Trial2.aspx?TrialID)= IRCT2017101636816N1. Accessed 7 March 2018.
4. Tochukuwa U. The effect of a counseling Intervention on Risk of IPV in Pregnant women in Nigeria. International Clinical Trial Registry Platform, WHO. 2017. [http://apps.who.int/ trialsearch/](http://apps.who.int/%20trialsearch/)Trial2.aspx?TrialID=PACTR201801002909246. Accessed 7 March 2018.
5. Dinmohammadi S, Kharaghani R. The effect of solution focused counseling on violence rate and quality of life of pregnant women at risk of domestic violence. International Clinical Trial Registry Platform, WHO. 2017. [http://apps.who.int/trialsearch/ Trial2.aspx?TrialID](http://apps.who.int/trialsearch/%20Trial2.aspx?TrialID)= IRCT2017040628352N4. Accessed 7 March 2018.
6. Hatcher A, Garcia-Moreno C. Addressing violence against pregnant women in antenatal care: testing an intervention in South Africa. International Clinical Trial Registry Platform, WHO. 2016. [http://apps.who.int/trialsearch/Trial2.aspx?TrialID =ISRCTN35969343](http://apps.who.int/trialsearch/Trial2.aspx?TrialID%20=ISRCTN35969343). Accessed 1 December, 2017.
