# Supplementary material for: Interventions for reducing and/or controlling domestic violence among pregnant women in low- and middle-income countries: a systematic review
Source: Syst Rev. 2019 Apr 2;8:79. doi: 10.1186/s13643-019-0998-4 (PMC6889323; doi:10.1186/s13643-019-0998-4)
Supplement: Supplementary file 4 — Quality appraisal of before and after studies. (DOCX 13 kb) [file 13643_2019_998_MOESM4_ESM.docx]

**Quality appraisal of before and after studies**

| **Criteria** | Turan et al., 2013 | Matseke & Peltzer 2013 | Krishnan et al., 2012 |
| --- | --- | --- | --- |
| 1. Is it clear in the study what the ‘cause’ is and what is the ‘effect’ (i.e. there is no confusion about which variable comes first)? | **Y** | **Y** | **Y** |
| 2. Were the participants included in any comparisons similar? | **Y** | **Y** | **Y** |
| 3. Were the participants included in any comparisons receiving similar treatment/care, other than the exposure or intervention of interest? | **N** | **N** | **N** |
| 4. Was there a control group? | **N** | **N** | **N** |
| 5. Were there multiple measurements of the outcome both pre and post the intervention/exposure? | **N** | **N** | **N** |
| 6. Was follow up complete and if not, were differences between groups in terms of their follow up adequately described and analyzed? | **N** | **Y** | **Y** |
| 7. Were the outcomes of participants included in any comparisons measured in the same way? | **Y** | **Y** | **Y** |
| 8. Were outcomes measured in a reliable way? | **UC** | **Y** | **UC** |
| 9. Was appropriate statistical analysis used? | **N** | **Y** | **N** |
| Total | **3/9** | **6/9** | **4/9** |
| **Overall Appraisal** | Small sample size, outcomes not clearly explained, statistical analysis not done. | High drop-out rates, confounders not controlled. | Pilot study, small sample size, and no statistical calculation. |

Y: Yes; N: No; UC: Unclear
