# Supplementary material for: Interventions for reducing and/or controlling domestic violence among pregnant women in low- and middle-income countries: a systematic review
Source: Syst Rev. 2019 Apr 2;8:79. doi: 10.1186/s13643-019-0998-4 (PMC6889323; doi:10.1186/s13643-019-0998-4)
Supplement: Supplementary file 5 — Table S1.Overview of studies in the review. (DOCX 49 kb) [file 13643_2019_998_MOESM5_ESM.docx]

| **Study References** | **Study Type** | **Sample size** | **Country/Setting** | **Intervention Details** | | | | | | | **Outcomes** | |
| --- | --- | --- | --- | --- | --- | --- | --- | --- | --- | --- | --- | --- |
|  |  |  |  | **Time** | **No.** | **F/U** | **Delivered by** | **Content** | **Mode** | **Description** | **DV** | **MH** |
| Cripe et al., 2010 | RCT | IG (104)  CG (100) | Peru | 30 min | 1 | 6 wks | Trained social workers |  | 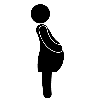 | Empowerment intervention consisting of abuse assessment, wallet-size referral card, 30 minute supportive counselling and education | NR | NS |
| Jones et al., 2013 | RCT | IG (238)  CG (240) | South Africa | 90-120 min | 4 | 7 wks | HIV counsellor |  | 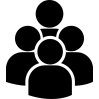 | PartnerPlus intervention emphasizing cognitive-behavioural skill building to improve communication, sexual negotiation, conflict resolution, STI/HIV prevention, PMTCT, use of condoms, and gender-relevant issues. | S | NR |
| Krishnan et al., 2012 | pre- and post-intervention | IG (20 dyads) | India | 3 hours | 8 | No | Study staff |  | 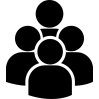 | Information of gender roles, women’s work, GBV, relationship skills training, family health promotion and healthy aging, intergeneration communication (only for MILs). | NC | NR |
| Matseke et al., 2013 |  | IG (230) | South Africa | 20 min | 1 | 3 mnth | Community workers |  | 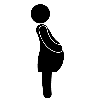 | The intervention was based on Dutton’s empowerment model and included supportive care, anticipatory guidance, guided referrals. | S | NR |
| Turan et al., 2013 |  | IG (134) | Kenya | NR | 1 | NR | Health clinic staff |  | 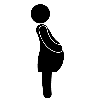 | Training to clinic staffs, screening, supported referrals and anti-GBV messages dissemination. | NR | NR |

Table S1 Overview of studies in the review

IG: Intervention Group; CG: Control group; Pink: Domestic violence related intervention, Blue: HIV related intervention; NS: Non-significant, NR: Not reported, S: Significant;

DV: Domestic Violence; MH: Mental Health
